# High‐Sensitivity Perovskite γ‐Ray Detectors Enhanced by Device Engineering Toward Energy Spectroscopy Imaging

**DOI:** 10.1002/advs.202503597

**Published:** 2025-07-12

**Authors:** Ken Qin, Jia‐He Zhang, Ruo‐Long Zhou, Xue Sun, He‐Xuan Wang, Jia‐Li Peng, Min‐Shu Zhang, Guan‐Hua Dun, Dan Xie, He Tian, Yi Yang, Tian‐Ling Ren

**Affiliations:** ^1^ School of Integrated Circuits and Beijing National Research Center for Information Science and Technology (BNRist) Tsinghua University Beijing 100084 P. R. China; ^2^ State Key Laboratory of Crystal Materials Institute of Crystal Materials Shandong University Jinan 250100 P. R. China

**Keywords:** γ‐ray detectors, perovskite, spectral enhenced imaging

## Abstract

Perovskite‐based detectors for γ‐rays have emerged relatively recently and have attracted limited attention. Although increasing efforts are being directed towards their development, the absence of an adaptive processor, systematic theoretical framework, and various applications limit its further development. In this paper, drawing inspiration from the commercial detectors, electrode configuration is improved to reshape the bias electric field, guide the effective signal carrier, and mitigate the noise from the side section. Through theoretical analysis, simulations, and experimental comparisons, the clear improvement of energy resolution from 7% to ≈5% is demonstrated, and the highest resolution of 1.9% is observed. Furthermore, to fill up the research framework of radiation, the definition of X‐ray sensitivity is extended to quantitatively describe the γ‐ray response, and assess the performance of γ‐ray detectors, reaching a sensitivity ≈10^5^ µC Gy_air_^−1^ cm^−2^. Based on these, a spectral‐enhanced imaging strategy is proposed to broaden the application of γ‐Ray detectors, where the spectral dimension of images is utilized to improve contrast and enhance imaging quality.

## Graphical abstract

Optimized electrode configuration in perovskite γ‐ray detectors achieves enhanced energy resolution from 7% to 1.9% by reshaping bias electric fields and reducing noise. A novel spectral‐enhanced imaging strategy is developed, utilizing spectral dimensions to improve image contrast and quality. This work demonstrates high sensitivity (~10⁵ µC Gy_air_⁻¹ cm⁻²) and establishes spectroscopic imaging applications for γ‐ray detection.

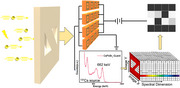

## 1 Introduction

Radiation detection and imaging has a broad application in industrial non‐destructive inspection,^[^ ^1^ ^]^ scientific research,^[^ ^2^ ^]^ medical treatment, and astronomy,^[^ ^3^ ^]^ which has increased the demand for next‐generation materials with better performance. However, there are several issues, such as the material cost,^[^ ^4^ ^]^ fabrication complexity,^[^ ^5^ ^]^ and the stringent operating conditions,^[^ ^6^ ^]^ restricting the development.^[^ ^7^ ^]^ Radiation detectors can be classified into scintillators and semiconductor detectors. Wherein, the scintillators transfer the radiation into visible light, such as the NaI and LaBr_3._ But an intrinsic non‐proportionality exists in this procedure, leading to measurement inaccuracies.^[^ ^8^, ^9^ ^]^ Semiconductor detectors directly convert the radiation into electrical signals, offering greater accuracy than the detector of scintillator‐based detectors. Semiconductor for radiation detection needs to satisfy two fundamental requirements: high absorbency and efficient conversion capability of high‐energy photons, along with large charge mobility and long lifetime. However, only a few kinds of semiconductors, like the Cd_x_Zn_1‐x_Te, HgI_2,_ and high‐purity Ge, meet the required detection capabilities. What makes it worse is the difficulty in production, environmental stability, and the demanding working condition, greatly hindering their commercializing.

Since the successful application of solar cells has brought perovskite materials into the spotlight of researchers, they have been explored for various other applications, such as light‐emitting diodes, catalysis,^[^ ^10^ ^]^ and detectors. As the next‐generation bandgap‐controllable semiconductor, perovskites, possess a high atomic number, making them suitable for radiation absorption.^[^ ^11^, ^12^ ^]^ Additionally, perovskites exhibit considerable stability, working condition adaptability, and straightforward solution‐based fabrication, which has been deemed as one of the most excellent candidates for new‐generation radiation detection.

Yakunin et al. demonstrated the γ‐ray photons detection of ^241^Am with an energy resolution of 35% by MAPbI_3_.^[^ ^13^ ^]^ He et al. achieved a high energy resolution of the ^137^Cs spectra with the modified melt‐growth CsPbBr_3_ perovskite, significantly improving the resolution to 3.8%,^[^ ^14^ ^]^ and even 1.8%.^[^ ^15^ ^]^ Zhao et al. improved the resolution of FAPbBr_3_ to 1.7% by surface‐defect‐passivation to the perovskite.^[^ ^16^ ^]^ These solution‐grown perovskites have been employed in γ‐ray spectroscopy detection due to their naturally smooth surfaces and low defect density. However, perovskites grown through natural solution methods exhibit uncontrollable morphology, variations in crystal size, and device‐to‐device inconsistencies, which limit their application in standardized production and practical scenarios. Furthermore, perovskite spectroscopic imaging remains challenging due to the low yield of crystals suitable for γ‐ray spectroscopy detection. In contrast, melt‐grown perovskite single crystals offer superior control over dimensions and uniformity, enabling homogeneous mass production through standard cutting and polishing procedures. Nevertheless, the interfaces created during cutting and polishing processes can introduce interfacial defects and lead to adverse effects such as dark current deterioration, which hinders the application of melt‐grown crystals in γ‐ray detection and imaging.

**Figure 1:**
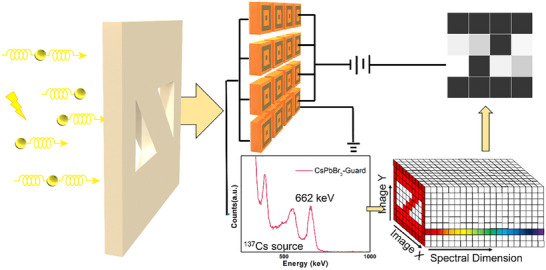
Schematic images of the spectral enhancing imaging by patterned Pb plate cutting off the γ‐ray. The perovskite device is fabricated with the asymmetric guard‐ring electrode and the spectral dimension information of the images are utilized to enhance the imaging and improve the quality.

Herein, we report a modified electrode design for CsPbBr_3_ perovskite, enabling the detection of the γ‐ray and exhibiting a dramatic improvement in the dark current, which can theoretically eliminate ≈30% of the current, covering most of the leakage current caused by cutting and polishing process, eventually improving the spectral resolution to 1.9%. We also innovatively combine energy spectra with radiation imaging, which can effectively eliminate the noise by energy resolution, greatly improving the quality of the imaging (**Figure** **1**).^[^ ^17^ ^]^ Finally, we assess the radiation‐stability of the perovskite detectors and the sensitivity of perovskite to the γ‐ray of 96 840.0 µC Gy_air_^−1^ cm^−2^. With the combination of the high‐quality perovskites and improved read‐out circuit, we believe the perovskites will become a more promising alternative of the γ‐ray detectors.

## 2 Results

### 2.1 Perovskite Growth and Characterizations

Melt‐grown Bridgman method is a simpler and faster strategy for preparing high‐quality perovskite crystals.^[^ ^18^ ^]^ Compared with conventional methods, solution‐grown methods suffer from the issue of low solubility in solution, the solution caused defects and contamination, which limit the size of the crystal and also hinder perovskite performance.^[^ ^19^ ^]^ Therefore, the Bridgman method has been adopted to grow CsPbBr_3_ perovskite single crystals in this work (Figure S1, Supporting Information).

**Figure 2:**
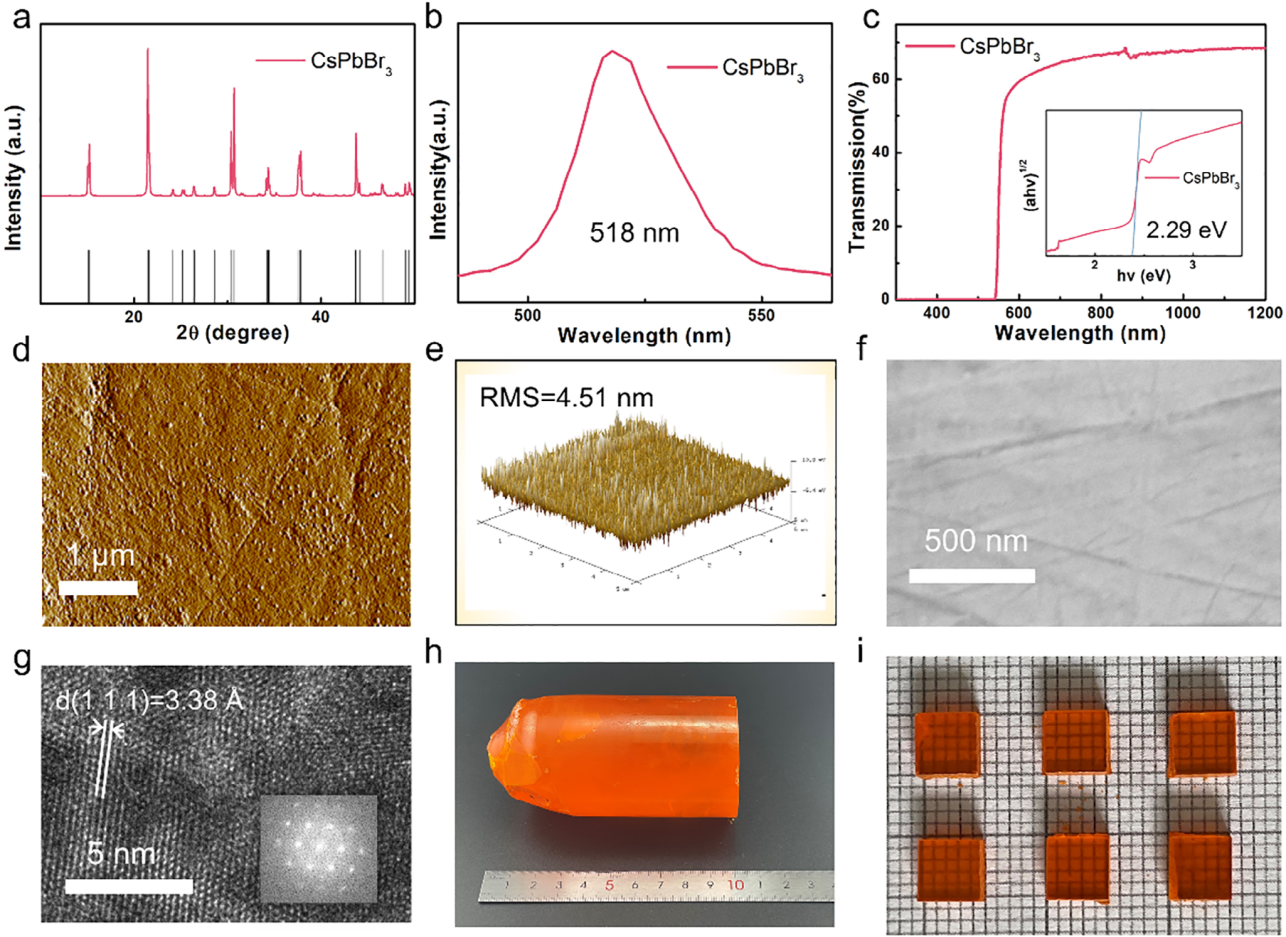
a) The XRD pattern of CsPbBr_3_ perovskites single crystal with the calculated standard patterns (Black). b) The PL result of perovskites. c) Optical transmission spectrum for perovskites (Insert: The Tauc Plot results and the bandgap energy by calculated). d,e) The AFM results of perovskites within 25 µm^2^ and the RMS. f) SEM result of perovskite surface after post‐treatment. g) High resolution TEM with selected area electron diffraction (insert). h,i) The pictures of CsPbBr_3_ perovskites single crystal after crystallization and after post‐treatment.

The X‐ray diffraction (XRD) patterns of as‐grown crystals exhibit a good material phase purity, while the peaks correspond well to the calculated standard results, which are shown in **Figure** **2**a. The crystal demonstrates photoluminescent emission peak at 518 nm under the excitation at 420 nm (Figure 2b), consistent with previously reported values.^[^ ^14^ ^]^ For a 2‐mm‐thick crystal, the optical transmittance exceeds 60% at the wavelength of 610 nm, and the bandgap energy is determined as 2.29 eV via Tauc plots^[^ ^14^ ^]^ (Figure 2c). Using the Hecht equation by analyzing the voltage‐dependent spectral response of the detectors, the µτ value for electrons was estimated to be ∼5 × 10⁻^3^ cm^2^ V^−1^ (Figure S2, Supporting Information). Furthermore, scanning electron microscopy (SEM) and atomic force microscopy (AFM) are employed to inspect the crystal surface, which acts as an important role in the signal transport. The polished crystal exhibits a root mean square (RMS) roughness of 4.51 nm, which is lower than that of the original crystal (Figure S3, Supporting Information) of 100 nm (Figure 2d‐f). High‐resolution transmission electron microscopy (HRTEM) confirms the single crystallinity and uniformity of the CsPbBr_3_, as illustrated in Figure 2g. The zone axis is determined to be the [0 1 ‐1] zone based on the TEM image simulation.

### 2.2 Electrode Design and Analysis

First, different electrode materials are utilized to form an asymmetric electrode configuration, based on the energy band structure of CsPbBr_3_. Compared to identical electrodes, the asymmetric ones can form Schottky junctions and the barrier potential to limit the dark current.^[^ ^14^, ^20^ ^]^ Specifically, materials with small work functions like Ag (4.26 eV) or Ga (4.3 eV) can form a higher Schottky barrier potential *ϕ_p_* under reversed bias, thereby reducing the dark current. In contrast, a material with a relatively high work function like Au (5.1 eV) is deposited on the opposite side, working as hole extraction layers. Given that the conduction band minimum and the valence band maximum for our CsPbBr_3_ perovskite to be ‐3.3 and ‐5.6 eV.^[^ ^21^ ^]^ The implementation of Ag electrodes was motivated by its combination of high charge carrier mobility, chemical inertness, and compatibility with standard industrial fabrication protocols. Although Ga was considered as an alternative electrode material, its high fluidity and chemical reactivity made it incompatible with stable device fabrication.

**Figure 3:**
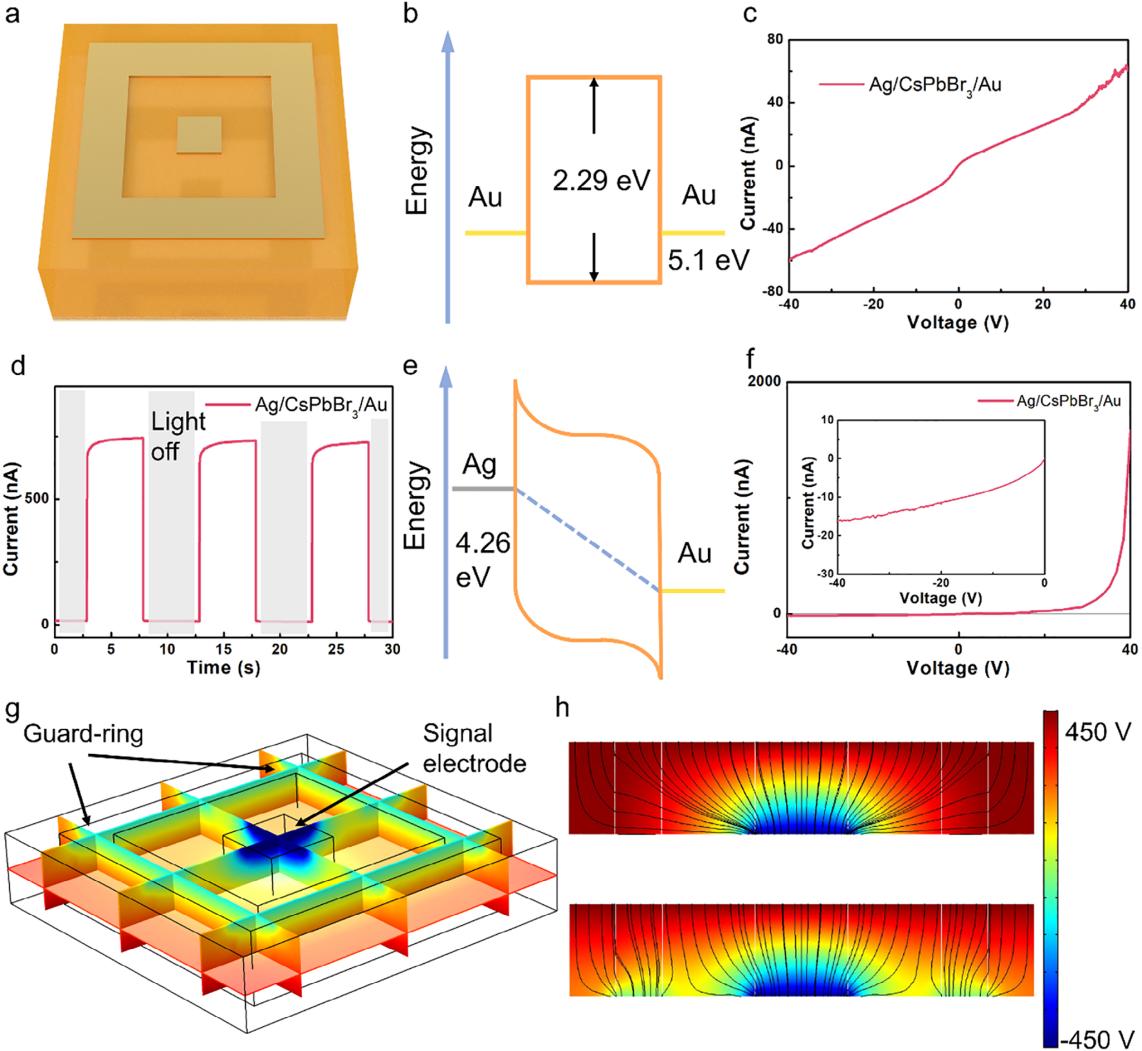
a) Schematic images of perovskites with asymmetric guard‐ring electrode. b,c) Band diagrams of traditional symmetric electrodes and the typical current to voltage (*I–V*) characteristic curve. d–f) Band diagrams of asymmetric electrode, and the I‐V, current to time (*I–t*) response of the perovskite devices. g,h) Simulation of the 3D electrical potential and the electrical filed line comparation from a sectional typical view, while the upper one is a traditional electrode and below is the distribution of our new design.

The comparison of current‐voltage further confirms the current‐limiting effect as shown in **Figure** **3**a‐f. The device with an asymmetric electrode shows a low level of current under an opposite bias voltage, as we expected, while the symmetry Au/Au device shows a rather higher current under the same bias voltage. Moreover, the asymmetric electrodes don't affect the photo‐response of the perovskite, either the response speed or sensitivity, as indicated in Figure 3d. The devices also exhibit a ‐3 dB bandwidth at 5 V of 2.0 kHz (Figure S6, Supporting Information), which is comparable with other perovskite devices.^[^ ^22^, ^23^ ^]^

Second, we engineered an optimized guard‐ring architecture that synergistically integrates with our asymmetric electrode design to address the critical challenges in charge collection uniformity and edge effects (Figure 3a, Figure S4, Supporting Information). This design reimagines conventional guard‐ring principles^[^ ^24^, ^25^, ^26^ ^]^ to match the unique requirements of our high‐performance detector structure. Crystal segmentation during detector fabrication creates surface states and structural defects at the cutting interfaces. These interface defects generate additional charge trapping centers and uneven field distributions that affect detector performance. Based on quantitative analysis of existing guard‐ring designs, we identified two key configurations: the side‐wall implementation that monitors carrier transport,^[^ ^24^ ^]^ and the electrode‐surface design that modifies the electric field distribution near the crystal edges. Integrating these design principles, we developed a guard‐ring structure optimized for our asymmetric electrode configuration.

The guard‐ring structure consists of an Au ring surrounding the pixelated Au electrode, with the Ag electrode area remaining unmodified. This configuration aligns with the distinct functions of our electrode materials: the Ag forms a large‐area Schottky contact for carrier collection, while the Au electrode serves as the signal readout layer. Through independent grounding of the guard‐ring, the structure effectively reduces edge‐related leakage current and normalizes the electric field distribution near the crystal boundaries, thereby maintaining the detection capabilities of the asymmetric electrode design.^[^ ^27^ ^]^

We also conduct a theoretical analysis to simulate the carrier collection differences between the traditional electrode and our new design. The simulation is performed based on the software COMSOL and the details are provided in METHODS.^[^ ^28^ ^]^ The overall electrical potential in 3D is exhibited in Figure 3g, while the distribution and the electric field line of the main cross‐section are depicted in Figure 3h. It is evident that the electric field lines and the current from the section side is directed toward to the guard‐ring electrode, as shown by the horizontal electrical filed weight in Figure S8 (Supporting Information). The result confirms that the noise signals from the side section defect are guided by the electrical field and absorbed by the guard‐ring structure, while the effective signal from the center is directed to the collecting electrode and remains unaffected.^[^ ^29^, ^30^ ^]^ From the electrical field distribution, it can be calculated that the guard‐ring design mainly absorbs the noise, with ≈60% of the effective signal is still collected by the Au signal‐collecting electrode.

### 2.3 γ Radiation spectral performance

**Figure 4:**
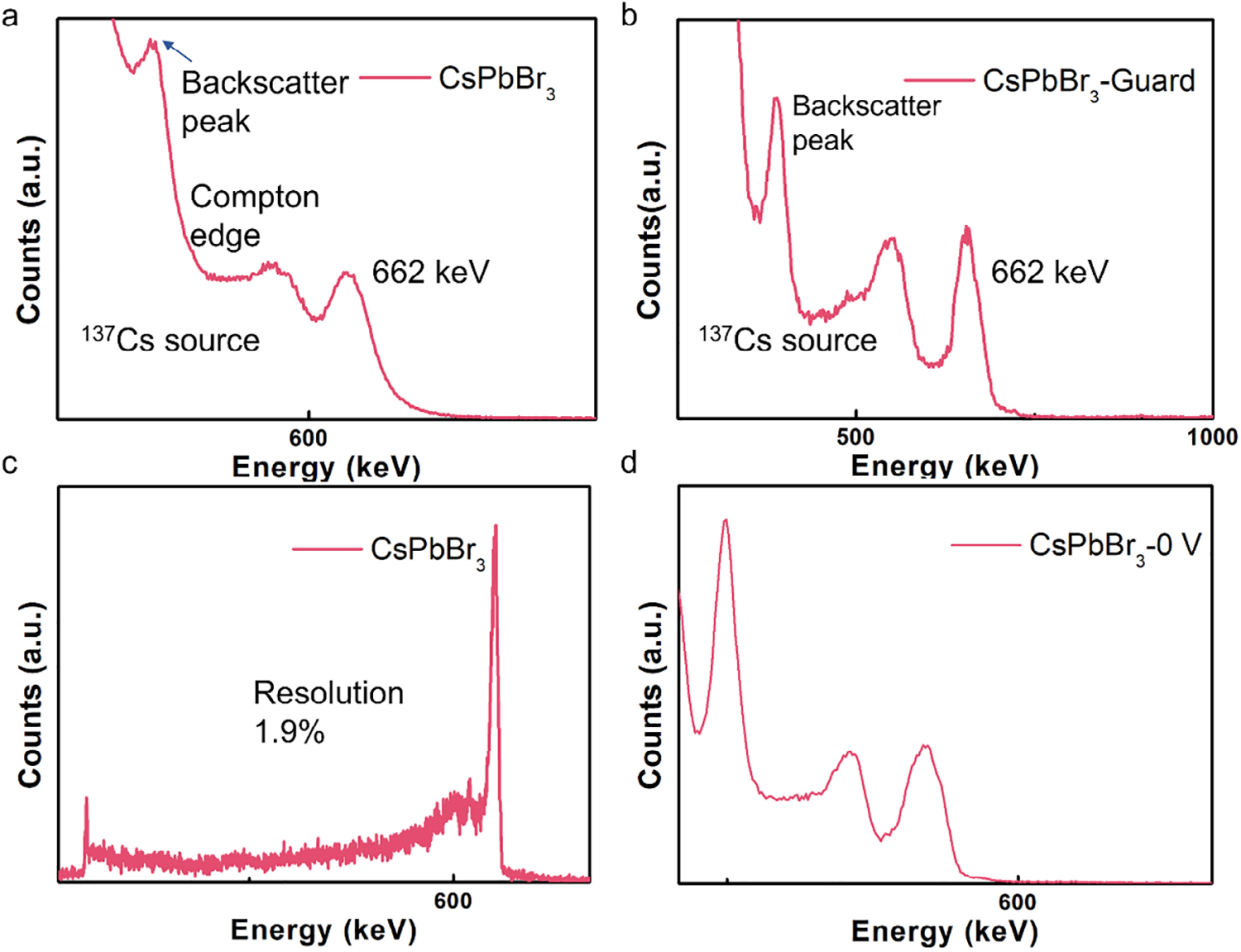
a,b) Energy‐resolved spectrum of ^137^Cs γ‐ray with a characteristic energy of 662 keV from perovskite devices with the traditional symmetric electrode and with asymmetric guard‐ring electrode, respectively. c) The spectrum of ^137^Cs γ‐ray from CsPbBr_3_ perovskite detectors with both asymmetric electrode and guard‐ring electrode. d) Energy‐resolved spectrum of ^137^Cs γ‐ray with a characteristic energy of 662 keV from perovskite devices under 0 V bias voltage.

To quantitatively assess the real effect of the new electrode design quantitatively, several perovskite detectors are fabricated based on CsPbBr_3_ from the same batch and of the same size, but with different electrode configurations. Compared with the asymmetric Au/Ag electrode, some of the detectors are deposited with the symmetric planar Au/Au electrode. Both sets of detectors are used to demonstrate the ^137^Cs γ‐ray spectrum, and the results are shown in the **Figure** **4**a,b. When exposed to the 662 keV ^137^Cs γ‐ray radiation, the symmetric detectors (Au/CsPbBr_3_/Au) without guard‐ring electrode show a spectroscopic response under a bias voltage of 900 V. Figure 4a displays the energy‐resolved spectrum of ^137^Cs with the backscatter peak, Compton edge and 662 keV main peak clearly defined,^[^ ^14^ ^]^ and an energy resolution ≈7.5%. Surprisingly, with the same condition, the detectors with guard‐ring exhibit a definitely better response to the same ^137^Cs γ‐ray source. As it can be seen in the Figure 4b, all the characteristic peaks mentioned above are clearly present in the same place, with each peak appearing sharper and more distinct. Moreover, the resolution also improves quantitatively from 7.5% to ≈5%.

When the detector is fabricated with both asymmetric electrode and guard‐ring electrode (Au‐guard/CsPbBr_3_/Ag). The highest energy resolution observed in our experiment reached 1.9%, as shown in Figure 4c. The performance above is attributed to the electrode design, the asymmetric electrode material and guard‐ring structure decrease the dark current and block the noise, thus making the signal prominent and collected efficiently.^[^ ^31^ ^]^

Furthermore, an interesting observation is made with the detector featuring our new electrode design. The detector is capable for self‐powered spectrum measuring as shown in Figure 4d, where the spectrum under ^241^Am and ^137^Cs γ‐ray radiation is obtained without a bias voltage. Although the spectrum has minimal differences and the main energy peak experiences a slight displacement, which we attribute to the absence of the drift electric filed provided by the bias voltage. The type of the γ‐ray source can be still distinguished by the whole spectrum. Generally, the self‐powered phenomenon is associated with the presence of the built‐in electric field. In this case, we attribute it to the asymmetric electrode design. The electrodes made of different kinds of materials form two homonymous Schottky junctions, which both contribute to the formation of a built‐in electric field and facilitate carrier collection even without the bias voltage.^[^ ^32^ ^]^ Additionally, the channel number at 0 V also is the lowest we've ever measured. Because the built‐in electric field from the asymmetric electrode is very weak, which has the same tendency with the experiment above and is evident our explanation about the relationship of the spectrum and bias voltage.

**Figure 5:**
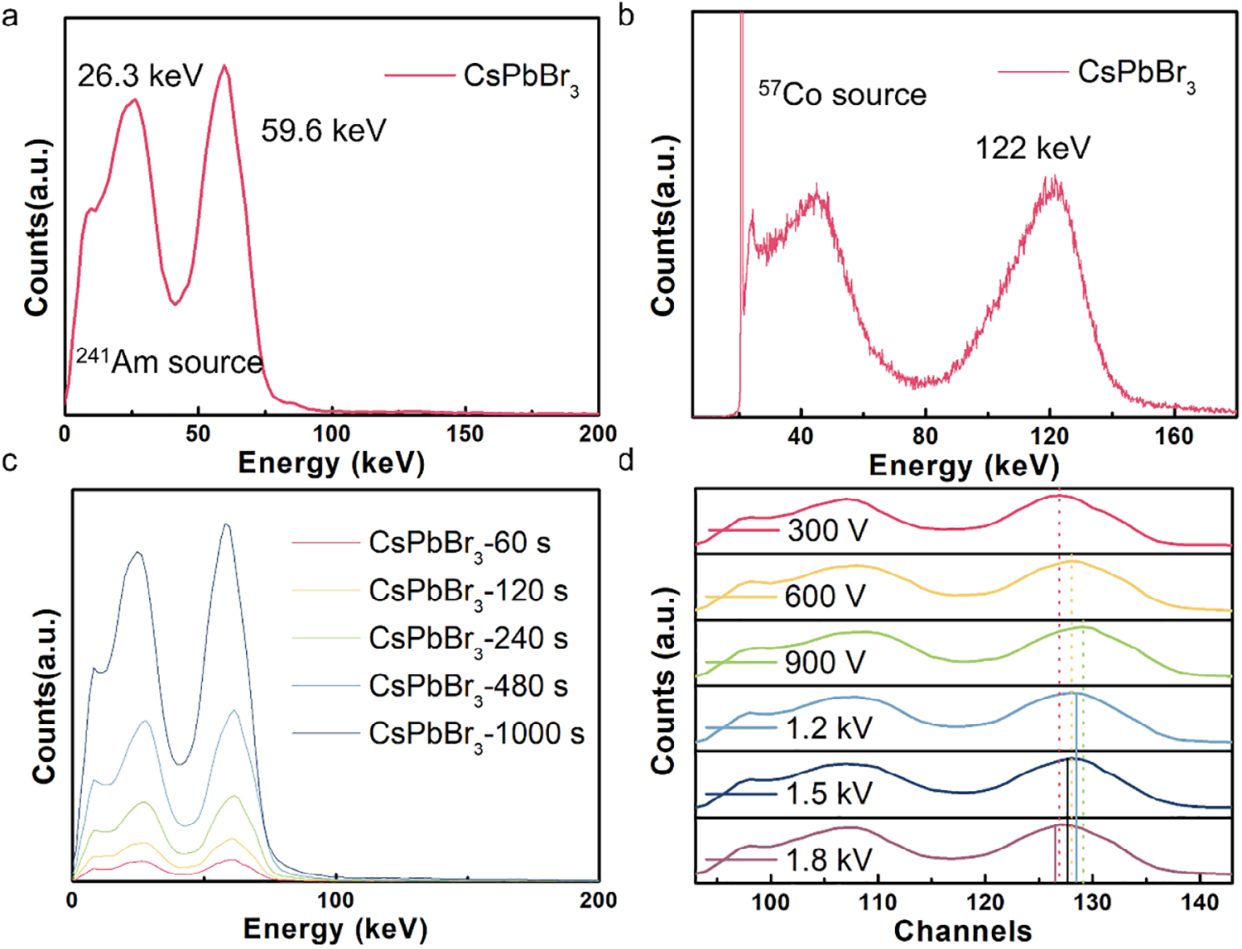
a,b) Energy‐resolved spectrum of ^241^Am and ^57^Co γ‐ray source. c) The stability of spectrum of CsPbBr_3_ perovskite detectors within different time over a whole period of 1000 s under ^241^Am γ‐ray source. d) Spectrum of ^241^Am γ‐ray acquired with different bias voltage.

Spectroscopic response of the asymmetric detectors to ^241^Am and ^57^Co γ‐ray radiation is also carried out. As shown in **Figure** **5**a,b, detectors exhibit a spectrum with 59.6 and 122 keV main peaks for ^241^Am ^[^ ^13^ ^]^ and ^57^Co ^[^ ^33^ ^]^ respectively, similar to the spectrum of other perovskite γ‐ray detectors from other groups.

Our perovskite detectors demonstrate excellent stable response over time as Figure 5c exhibits. The figure illustrates the relation between the counts number and signal collection time. During a continuous period of time, the count rate and collection time remain a stable ratio, and the peak position remain unchanged. Moreover, a spectrum of γ‐ray usually requires several hours to complete the spectra accumulation in general, some may even take days. As for the perovskite detectors, this process is also reported to consume hours by the symmetric electrodes. However, despite the guard‐ring may block some signals, the signal collection occurs at a relatively high speed. Within minutes, the spectrum begins to take shape. After several quarters, the accumulation of counts is typically complete, demonstrating its high collection efficiency.

The relationship between the spectrum and the bias voltage is also studied using different detectors with separated bias voltage. From Figure 5d we can see that the shape of the spectrum or the energy resolution doesn't show significant change under different bias voltage. However, the channel number of the main peak has some subtle shifts. The channel number corresponding to the main energy peak increases with the bias voltage. After reaching the peak at 900 V, it begins to decrease, which is consistent to the rated voltage to get the best resolution, and the channel corresponds as others reported. We attribute this phenomenon to the drift electric field and ion migration. The channel number is directly related to the energy of the signal carrier by direct ratio. When the bias voltage is relatively low, the carrier gains drift energy from the electric field. Therefore, the channel number increases with a higher carrier energy from higher bias voltage. However, when the bias voltage becomes too large, the ion migration of the perovskite intensifies, causing a buildup of charged ions around the electrode.^[^ ^34^ ^]^ The interaction between the carrier and the ion leads to the energy loss of the signal and the decrease of the channel number.

### 2.4 γ Radiation Response Performance

The photon‐current response of the perovskite detectors to γ‐ray radiation is also investigated. During operation, detectors are exposed to all kinds of interference and noise from the atmosphere. Even in outer space, there are substantial amount of electromagnetism radiation. Therefore, in order to make sure the detectors can work properly in the ambient environment, it is crucial for a detector to have a clear and high‐sensitivity response to trace radiation, even when covered with noise, to ensure proper operation in the ambient environment.^[^ ^35^ ^]^ However, most research group focuses primarily on the property related to the spectrum and neglect the response sensitivity of the detectors. There has been limited effort in defining and measuring sensitivity, and as a result, the definition of γ‐ray sensitivity remains unclear. Some groups define it as the minimum of the detectable and distinguishable radiation rate.^[^ ^36^ ^]^ G. Heusser uses the activity of the radiation source to define the sensitivity, with the unit of Bq/Kg.^[^ ^37^ ^]^ J. A. Cooper refines this further, defining the sensitivity as the ratio of the counts number and gamma radiation and is given by

(1) $$R=\frac{N_{m}}{\varepsilon \cdot f\cdot t}$$

where the *N_m_* is the peak counts detectable after background subtraction, ε is the detector efficiency for measuring the γ‐ray of interest, *f* is the dosage of γ‐ray radiation and t is the counting time.^[^ ^36^ ^]^

However, these definitions are not so accurate or complex to measure. Therefore, we proposed extending the concept of sensitivity of the X‐ray to the field of γ‐ray,^[^ ^38^ ^]^ which is given by

(2) $$R=\frac{I_{p}-I_{d}}{D\times S}$$

where the *I_p_* and *I_d_* refer to the current under radiation in the ambient atmosphere and the current without radiation respectively. The *D* is the dose rate of γ‐ray in air and *S* represents the device effective area. This definition substitutes the current for the count number, making it easier to measure and also more accurate. And the other part of the equation agrees with the previous, that the dose rate and the effective area are in accordance with the received dosage and counting time.

**Figure 6:**
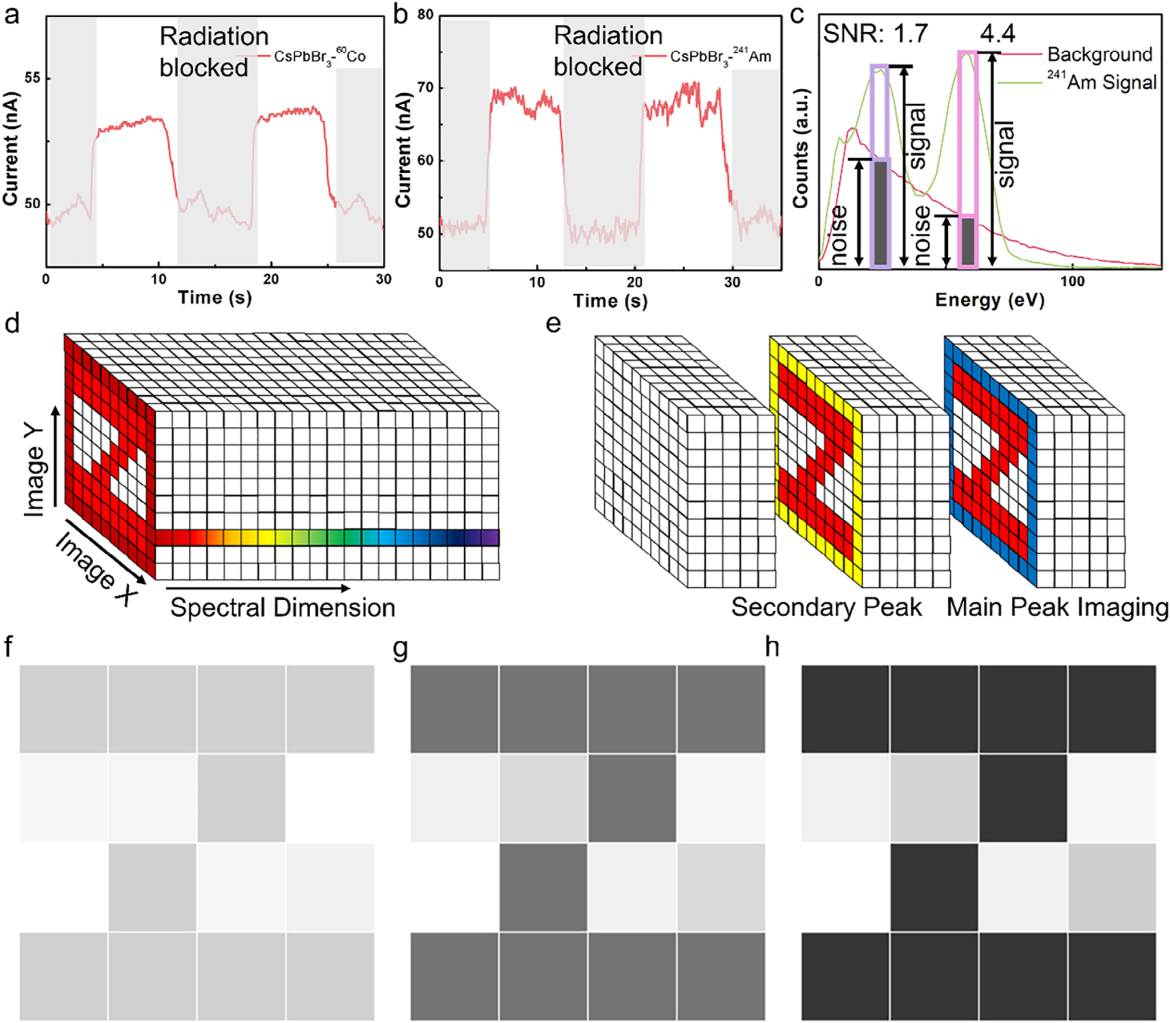
a,b) The I‐t response of the perovskite detectors under ^60^Co, ^241^Am γ‐ray. c) The filtering methods of spectral enhanced imaging and the signal to noise of each mode. d,e) Schematic picture of the spectral enhanced imaging. f–h) The pictures from directly imaging, spectral enhanced imaging by secondary and main energy peak, respectively.

We measure the sensitivity of our detectors to the γ‐ray with the help of a thick plate of lead (Pb). The current of the detectors is monitored by the digital semiconductor characterization system and the dose rate of the low activity, while the γ‐ray source is measured by the γ‐ray dose rate equivalent monitor. The detector operates under a bias voltage of 10 V from the semiconductor characterization system, exposed to the radiation of the γ‐ray source. The Pb plate is positioned between the detector and the γ‐ray source, acting as a switch. When placed, the Pb plate efficiently blocked radiation, allowing the detectors to only register radiation when it was not in position. This setup enabled us to generate a periodic current‐time diagram and calculate the sensitivity of the detectors. The γ‐ray response is shown in the **Figure** **6**a,b. As we can see, the current change is obviously to the switch of the radiation. Although the signal‐noise ratio is not so ideal, it is within our expect due to the low dose rate of the γ‐ray source, and it is fortunately still distinguishable. Furthermore, the sensitivity of the detectors is obtained by the formula above, with the device effective area of 0.09 cm^2^ and dose rate of 0.788 nGy_air_ s^−1^ (^241^Am), 0.218 nGy_air_ s^−1^ (^60^Co). The sensitivity is 35 266.7 µC Gy_air_^−1^ cm^−2^ to ^241^Am γ‐ray and 96 840.0 µC Gy_air_^−1^ cm^−2^ to ^60^Co. Our detectors show a high sensitivity to the γ‐ray, which ensure their detect ability against the noise signal in the ambient atmosphere. As shown in Table S1 (Supporting Information), the key metrics of the asymmetric detector are benchmarked against other commercial and perovskite‐based γ‐ray detectors. Notably, the device operates without requiring excessively high bias voltages and supports self‐powered detection at 0 V, while maintaining high energy resolution and sensitivity. In terms of stability, no performance degradation was observed even after cumulative irradiation up to 1000 Gy. In addition, it effectively resolves spectral peaks from multiple radionuclides, highlighting its applicability in a range of detection scenarios.

### 2.5 Spectral Enhanced Imaging

The spectral is the third dimension of images. Distinct from the spatial domain, the spectrum contains significantly more information. Spectral imaging has already seen widespread application in remote sensing and medical biomedical engineering, primarily through wavelength scanning, which focuses on visible light.^[^ ^17^, ^39^ ^]^ Herein, we proposed energy spectral enhanced imaging, which relies on radiation. Similar to medical X‐ray imaging, this technique reflects differences in the intensity of received radiation and is enhanced by spectral filtering (Figure 6d,e).

When the γ‐ray irradiates a thick and high‐atomic number object, the γ‐ray will be absorbed with only a small fraction can penetrate. As a result, pixels corresponding to the subject will receive less radiation, while other pixels will receive the full γ‐ray radiation, creating a contrast between the signal and the subject image. However, one of the most important challenges during the imaging is the processing and filtering of noise, which may come from the ambient atmosphere. This is especially problematic when dealing with high levels of radiation noise, where imaging without proper filtering can become significantly more difficult.

We demonstrate enhanced imaging through energy spectral techniques, using perovskite detectors array and γ‐ray sources. Three noise‐processing methods were compared. The first method is similar to conventional imaging, where all signals are accepted. The second and third methods use spectral filtering to isolate signals from either a single low‐power peak channel or the main energy peak channel, respectively (Figure 6c). The imaging results are shown in the Figure 6f–h. As seen, the images filtered by the energy spectrum are noticeably clearer compared to the conventional method, with the signal‐to‐noise ratio (SNR) increasing from 1.0 to 1.7 and 4.4. Because the energy spectral helps remove some influence from the noise. The main energy peak produces a superior image because the radiation from the ambient atmosphere typically has lower energy, concentrating in the lower channels. Therefore, by utilizing energy spectral filtering, the imaging quality can be significantly enhanced.

## 3 Discussion

In conclusion, we develop a new electrode design for perovskite γ‐ray detection and demonstrate the comparison of the γ‐ray energy resolution between traditional electrodes and the new ones. Our new electrode design mitigates the effects of crystal boundary defects and optimizes the electric field distribution. The detector exhibits the energy diagram of ^137^Cs, ^57^Co, and ^241^Am γ‐ray, and the new electrode also enhances the energy resolution to the highest 1.9%. Using energy filtering, we also demonstrate the potential of energy‐spectral‐assisted imaging, which significantly increases the contrast ratio of the resulting images. Moreover, we suggest the definition of sensitivity and extend the X‐ray sensitivity formula to the γ‐ray detection field. We measure the sensitivity of our device, which reaches 96 840.0 µC Gy_air_^−1^ cm^−2^ under the radiation of ^60^Co γ‐ray. We believe this work can pave the way for further applications of perovskite materials and advance the development of γ‐ray detection technologies.

## 4 Experimental Section

### Perovskite Growth and Process

The CsPbBr_3_ perovskites were prepared by melt‐grown Bridgman methods. High‐purity PbBr_2_ (5 N) and CsBr (5 N) were purchased from Aladdin Chemistry Co. Ltd and used without further purification. A stoichiometric mixture of PbBr_2_ and CsBr (mole ratio 1:1) was loaded into a silica ampule. The ampule was then evacuated to 4 × 10^−4 ^Pa and sealed with an oxyhydrogen flame. The sealed ampule was subsequently transferred to a pit furnace and heated to ≈600 °C and slowly cooled down with temperature controller to get the orange color CsPbBr_3_ polycrystals.

The CsPbBr_3_ single crystals were obtained with the polycrystal grown above by a homemade vertical Bridgman furnace. The hot furnace zone was set ≈600–800 °C and the cold zone was set to be 200–400 °C. The temperature gradient near the interface was adjusted to 15–30 °C cm^−1^ near the melting point. And the CsPbBr_3_ polycrystals were transferred within a silica ampule into the Bridgman furnace, heated at 580–600 °C for 6 h. The ampule was slowly moved downward toward the cold zone with a speed of 0.2‐1 mm h^−1^. Finally, the temperature of the furnace was cooled to room temperature slowly after the crystallization finished.

The as grown CsPbBr_3_ crystals were cut into 2 mm thick wafers with STX‐202A diamond wire cutting machine and polished with 7000 mesh sandpaper using acetone as a lubricant. In the final step, the residue on the surface of the sample was removed by the washing of toluene.

### Electrode Fabrication

The Au electrode was prepared by electron‐beam evaporation (DE400), with a thickness of 80 nm and a deposition rate of ≈1 Å s^−1^. The guard‐ring pattern was defined using a high‐precision shadow mask fabricated via laser etching, with a minimum feature size of 100 µm. The mask was designed to produce the following geometry: a central pixel electrode with a width of 0.5 mm, a surrounding square‐loop guard ring also 0.5 mm wide, and a 1 mm gap between the pixel and the guard ring. The Ga electrode was brushing liquid Ga metal on the surface of the crystal. And the Ag electrode was prepared by thermal evaporation or silver paste. The signal electrodes were connected by Ag wire to the printed circuit board and then to the outer collection circuit. While the guard‐ring electrode was also connected to the printed circuit board and then to the ground by alligator clip wire.

### Perovskite Characterization

Atomic force microscope (AFM, Bruker Dimension Icon) and scanning electron microscope (SEM, Cael Zeiss Microscopy, Merlin) were used to characterize the morphology of perovskite.

The structure of the CsPbBr_3_ was measured by X‐ray diffraction (XRD, Bruker D8). The sample for High Resolution Transmission Electron (HRTEM) was prepared by Focus Ion Beam (FIB) (ZEISS, Crossbeam 340) from the internal crystals. The crystallinity and the uniformity of the perovskite was investigated by the HRTEM (JEOL, JEM F200).

The photo properties of the materials were analyzed by Raman Spectrometer (LabRAM HR Evolution HORIBA Jobin Yvon) (532 nm 50 mW), to get the photoluminescence of the film. The photoluminescence of the perovskite was characterized by Fluorescence Spector (Edinburgh Instruments, EI, FLS920). The photo transmission and absorption were measured by UV‐vis‐IR Spectrophotometer (Perkin Elmer, Lambda1050+).

### Electrical Properties and Detectors Performance

Semiconductor characterization system (Keithley, 2636B and 4200) was used to test the electrical properties of the devices. And the γ radiation response was also measured by this system, with the bias voltage of 10 V applied from the system.

The γ spectral was demonstrated by Ortec Spectral System, including Ortec480 pulser, Ortec672 spectroscopy amplifier, Ortec928 combination MCB, and Ortec556 high‐voltage power supply. The perovskite devices were placed in a working shield box and connected to the preamplifier from Imdetek Co. Ltd, ShanXi, China. The γ‐ray sources employed were 9.14×10^3 ^Bq ^137^Cs 662 keV, 8.99×10^4 ^Bq ^241^Am 59.5 keV, 5.34×10^3 ^Bq 57Co 122 keV, supplied by the China Institute of Atomic Energy.

### Simulation

The simulation was based on the COMSOL Multiphysics software, the size of the perovskite model was 4: 4: 1, close to the real size. And the material properties include the dielectric constant, conductivity, carrier mobility, carrier life and so on come from experiments and article reports. The bias voltage applied by the electrode was set as 900 V and the guard‐ring was connected to 0 V as ground.

### Spectral Enhanced Imaging

A plate made of Pb with “Z” size hollowed out was prepared to cut off the γ radiation, placed between the device and the γ source. One perovskite was used to measure the spectral of the γ source from different positions behind the “Z” plate to simulate the imaging process, by shifting the perovskite devices. Every spectral and the spectral with background noise were obtained within the same time and from the same parameter. After the single channel filtering and normalization, the intensity of the signal was transferred into grayscale and formed the images.

## Conflict of Interest

The authors declare no conflict of interest.

## Author Contributions

K.Q., J.‐H.Z. and R.‐L.Z equally contributed to this work. K.Q. and J.‐H.Z. conceived the experiments. R.‐L.Z. and K.Q. fabricated the devices and characterized detector performance. X.S. synthesized, characterized, and grew the single crystals. H.‐X.W., J.‐L.P. and K.Q. carried out the theoretical simulation. All authors discussed the results and commented on the manuscript.

## Supporting information



Supporting Information

## Data Availability

The data that support the findings of this study are available from the corresponding author upon reasonable request.
