# Supplementary material for: High‐Sensitivity Perovskite γ‐Ray Detectors Enhanced by Device Engineering Toward Energy Spectroscopy Imaging
Source: Adv Sci (Weinh). 2025 Jul 12;12(34):e03597. doi: 10.1002/advs.202503597 (PMC12442693; doi:10.1002/advs.202503597)
Supplement: Supplementary file 1 — Supporting Information [file ADVS-12-e03597-s001.docx]

Support Information


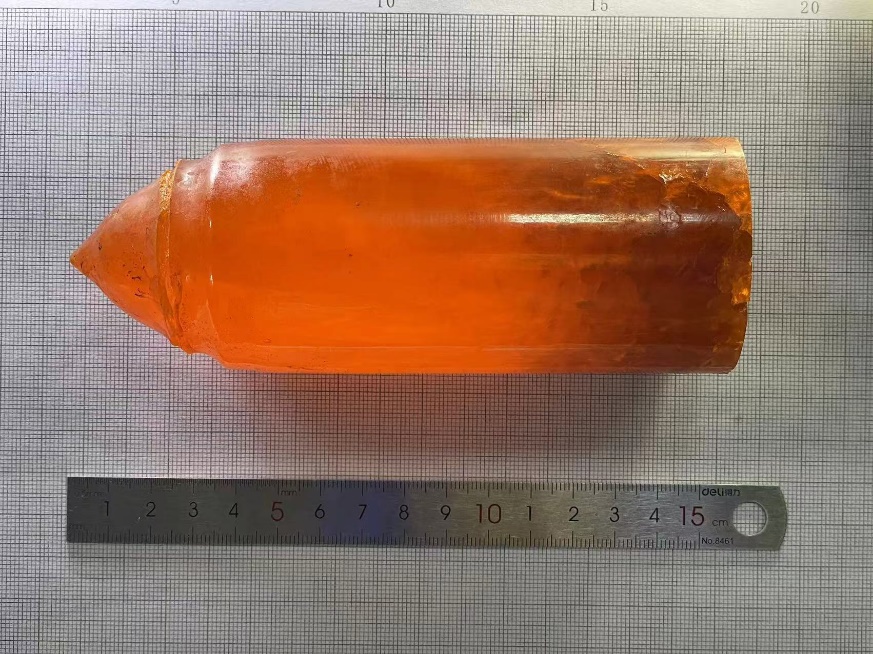


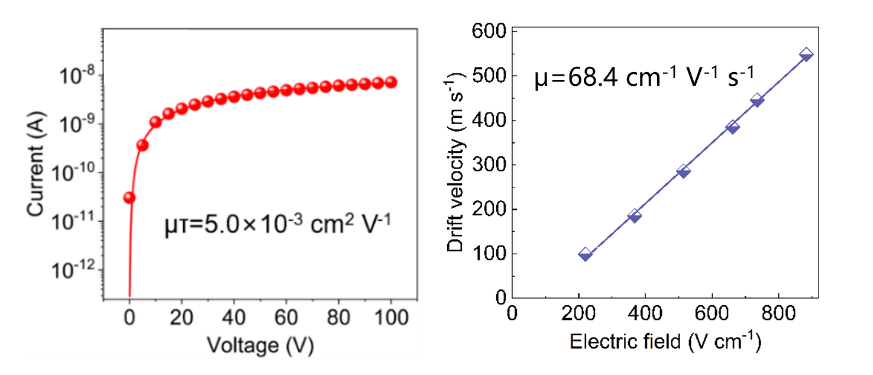
Fig.S1 Melt-grown CsPbBr_3_ perovskites crystal ingot of 15 cm length, right after the crystallization.

Fig.S2 The photocurrent fitting curve of the CsPbBr_3_ crystal according to the Hecht equation


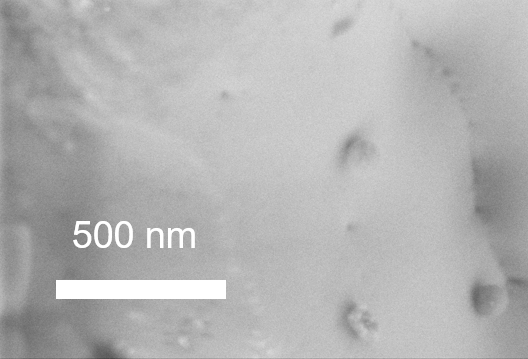


Fig.S3. SEM surface of the single crystal without post-processing, such as polishing and washing.


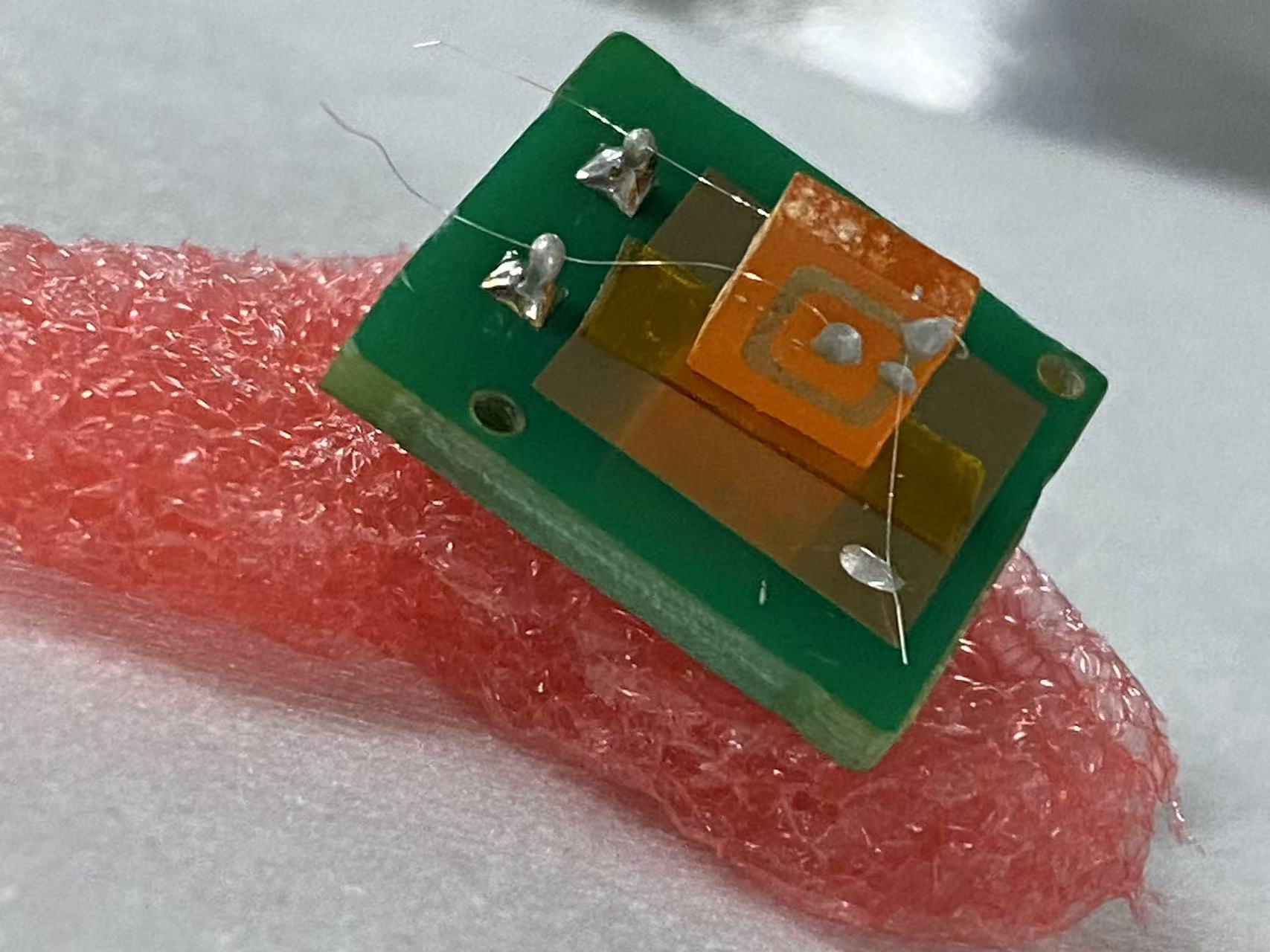


Fig.S4 The perovskite device with Au Gurad-ring electrode. The central signal electrode is connected to the outer read-out circuit and the guard-ring electrode is connected to the Au substrate and further connected to the Ground.


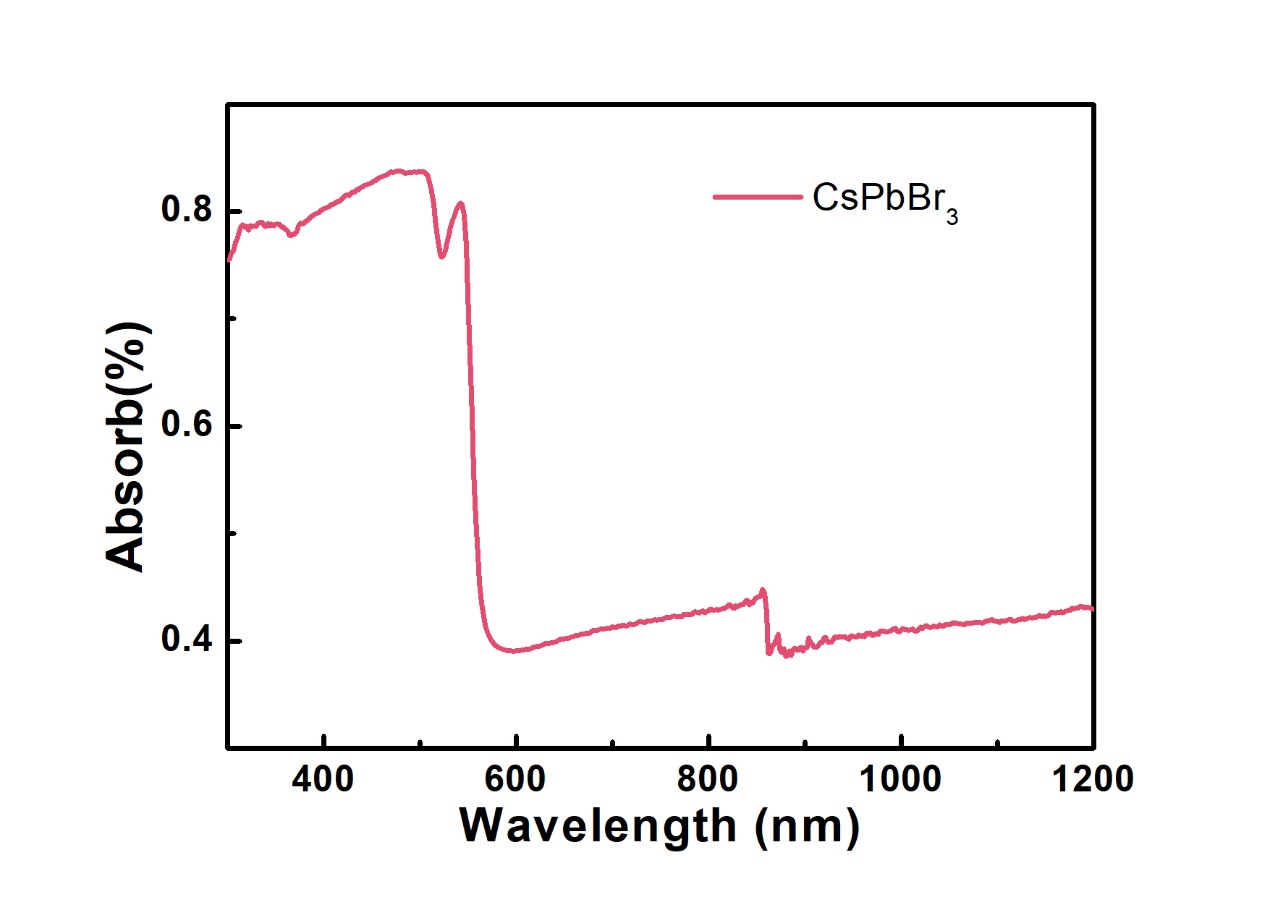


Fig.S5. Optical absorption spectrum of CsPbBr_3_ perovskite single crystal device.


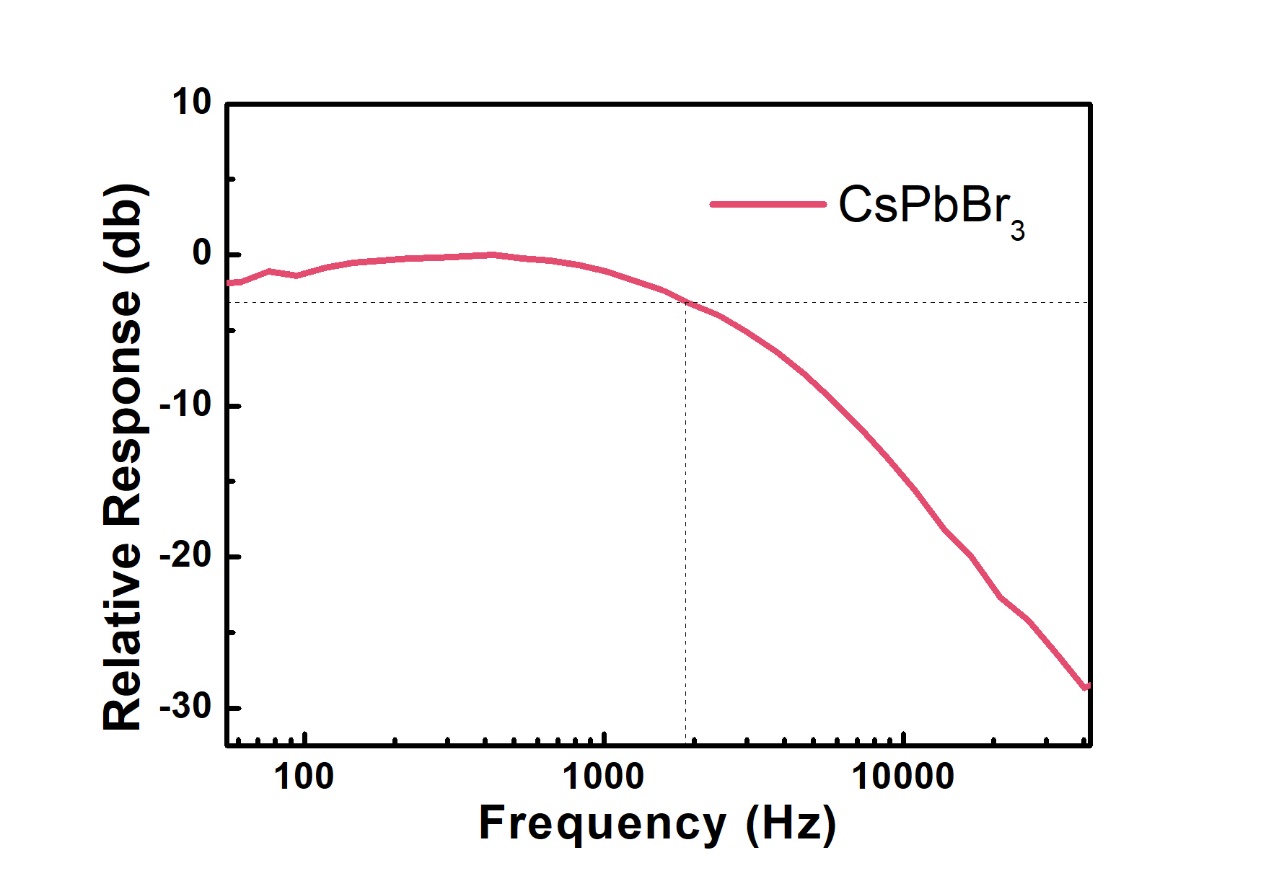


Fig.S6. -3db bandwidth about 2 kHz of the perovskite device at bias voltage of 5 V.


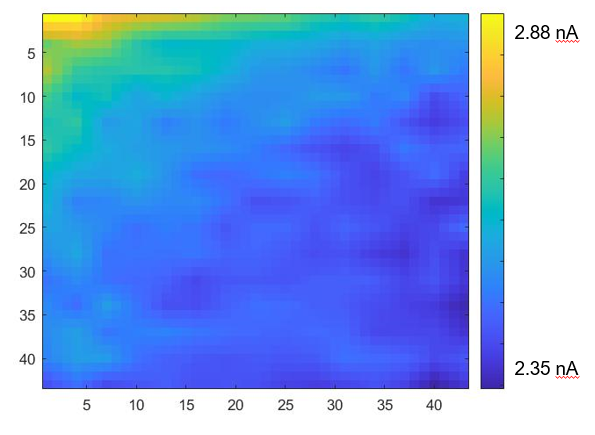


Fig.S7. Photo-response mapping by subtle laser light scan through the crystal surface to characterize the response uniformity.


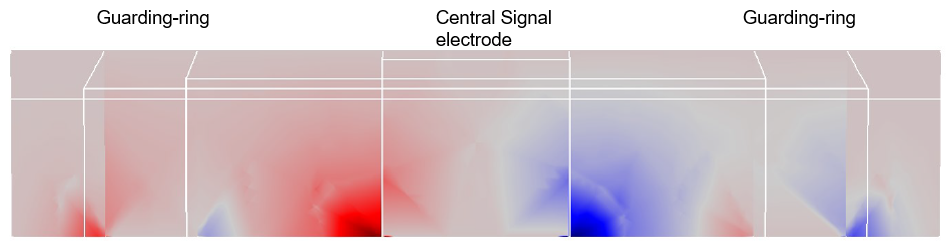


Fig.S8. The vertical weight of the electric filed of the device with guard-ring electrode by COMSOL simulation. While the red represent the toward the right, the blue represent the weight toward the left, exhibiting the distribution effect of the guard-ring..


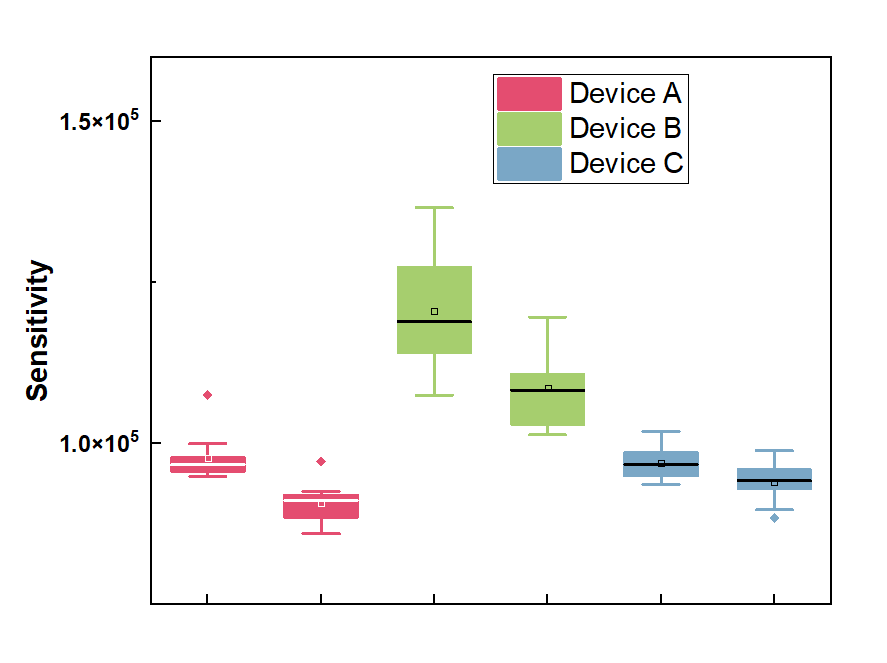


Fig.S9. The sensitivity measuring of three devices by 10 times. And the sensitivity under same condition of them after a month.

Table. S1 Performance comparison table of various γ-Ray detectors

| Detector Type | Bias Voltage | Energy Resolution | Sensitivity  (μC·Gy_air_⁻¹·cm⁻²) | Stability | Radiation source | ref |
| --- | --- | --- | --- | --- | --- | --- |
| HPGe | 3700V | 0.139%  (@1.33MeV) | NA | ＞18 months | ^137^Cs, ^57^Co, ^60^Co, ^133^Ba | (1, 2) |
| CZT | 1600V | 0.9%  (@662keV) | NA | ＞18 months | ^137^Cs, ^57^Co, ^60^Co,^241^Am, ^133^Ba, | (3, 4) |
| MAPbI_3_ | 50V | 6.8%  (@122keV) | 1400 | NA | ^57^Co,^241^Am | (*5, 6*) |
| CsPbBr_3_ | 500V | 1.4%  (@662keV) | NA | 18 months | ^137^Cs,^57^Co, ^241^Am | (7) |
| CsPbBr_3_ | 0 V / 900 V | 1.9 %  (@662keV) | 96,840 | ＞1000 Gy radiation | ^137^Cs, ^57^Co, ^60^Co,^241^Am | This Work |

**REFERENCES**

1. P. Sangsingkeow, K. D. Berry, E. J. Dumas, T. W. Raudorf, T. A. Underwood, Advances in germanium detector technology. *Nuclear Instruments and Methods in Physics Research Section A: Accelerators, Spectrometers, Detectors and Associated Equipment* **505**, 183-186 (2003).

2. N. Hafızoğlu, Efficiency and energy resolution of gamma spectrometry system with HPGe detector depending on variable source-to-detector distances. *The European Physical Journal Plus* **139**, 134 (2024).

3. F. Zhang, Z. He, C. E. Seifert, A Prototype Three-Dimensional Position Sensitive CdZnTe Detector Array. *IEEE Transactions on Nuclear Science* **54**, 843-848 (2007).

4. M. D. Alam, S. S. Nasim, S. Hasan, Recent progress in CdZnTe based room temperature detectors for nuclear radiation monitoring. *Progress in Nuclear Energy* **140**, 103918 (2021).

5. F. Qin, R. Zhao, W. Zhu, X. Tang, N. Deng, J. Wu, Z. Jiao, in *2023 IEEE International Conference on Manipulation, Manufacturing and Measurement on the Nanoscale (3M-NANO)*. (2023), pp. 323-326.

6. Y. He, W. Ke, G. C. B. Alexander, K. M. McCall, D. G. Chica, Z. Liu, I. Hadar, C. C. Stoumpos, B. W. Wessels, M. G. Kanatzidis, Resolving the Energy of γ-Ray Photons with MAPbI_3_ Single Crystals. *ACS Photonics* **5**, 4132-4138 (2018).

7. Y. He, M. Petryk, Z. Liu, D. G. Chica, I. Hadar, C. Leak, W. Ke, I. Spanopoulos, W. Lin, D. Y. Chung, B. W. Wessels, Z. He, M. G. Kanatzidis, CsPbBr_3_ perovskite detectors with 1.4% energy resolution for high-energy γ-rays. *Nature Photonics* **15**, 36-42 (2021).
